# Plasma heavy metal levels correlate with deregulated gene expression of detoxifying enzymes in osteoporotic patients

**DOI:** 10.1038/s41598-023-37410-8

**Published:** 2023-06-30

**Authors:** V. V. Visconti, B. Gasperini, C. Greggi, B. Battistini, A. Messina, M. Renzi, K. Bakhtafrouz, R. Iundusi, A. Botta, L. Palombi, U. Tarantino

**Affiliations:** 1 Department of Clinical Sciences and Translational Medicine, University of Rome Tor Vergata, Via Montpellier 1, 00133 Rome, Italy; 2 Department of Biomedicine and Prevention, University of Rome Tor Vergata, Via Montpellier 1, 00133 Rome, Italy; 3 Department of Orthopaedics and Traumatology, “Policlinico Tor Vergata” Foundation, Viale Oxford 81, 00133 Rome, Italy; 4 University “Nostra Signora del Buon Consiglio”, Tirana, Albania

**Keywords:** Pathogenesis, Risk factors

## Abstract

Heavy metal levels appear to be associated with low bone mineral density (BMD) and the consequent osteoporosis risk, but the relationship with the disease has not been clearly defined. The altered expression pattern of numerous genes, including detoxifying genes, seems to play a pivotal role in this context, leading to increased susceptibility to several diseases, including osteoporosis. The purpose of this study is to analyse circulating heavy metals levels and the expression of detoxifying genes in osteoporotic patients (OPs, n = 31), compared with healthy subjects (CTRs, n = 32). Heavy metals concentration in plasma samples was determined by Inductively Coupled Plasma Mass Spectrometry (ICP-MS), and the subsequent expression analysis of *NAD(P)H quinone dehydrogenase 1* (*NQO1*), *Catalase* (*CAT*), and *Metallothionein 1E* (*MT1E*) genes in Peripheral Blood Mononuclear Cells (PBMCs) was assessed by real-time polymerase chain reaction (qRT-PCR). Copper (Cu), mercury (Hg), molybdenum (Mo) and lead (Pb) were found to be significantly higher in the plasma of OPs compared to CTRs. Analysis of the expression levels of detoxifying genes showed a significant decrease in *CAT* and *MT1E* in OP group. In addition, Cu correlated positively with the expression levels of both *CAT* and *MT1E* in CTRs group and *MT1E* in OPs. This study shows an increased circulating concentration of certain metals combined with an altered expression pattern of detoxifying genes in OPs, highlighting a novel aspect to be investigated in order to better characterize the role of metals in the pathogenesis of osteoporosis.

## Introduction

Heavy metals such as Lead (Pb), Mercury (Hg), Arsenic (As), and Cadmium (Cd) have been classified by the World Health Organization (WHO) as global environmental pollutants that require constant monitoring given their numerous adverse health effects^1,2^. In fact, exposure to these pollutants can affect cardiovascular, endocrine, respiratory, reproductive, and immune systems^3^. Heavy metals are also considered capable of impairing the functionality of several proteins, and modulating the expression pattern of numerous genes, altering physiological processes and thus leading to increased susceptibility to the development of certain diseases^3^. The chemical properties of heavy metals allow them to interact with the cell's macromolecules, leading to their oxidative deterioration^4^. According to literature data, long-term exposure to heavy metals and their bioaccumulation can induce oxidative stress and also lead to dysregulation of detoxifying genes expression levels, which play a primary role in the elimination of Reactive Oxygen Species (ROS)^5^. It has been reported that Pb and Cd induce lipid peroxidation, impairing the functionality of key cellular anti-oxidant mechanisms by decreasing the activity of superoxide dismutase (SOD), catalase (CAT), and glutathione (GSH) levels^6,7^. According to further studies, in addition to Pb and Cd, As, Hg and Nickel (Ni) are also responsible for redox reactions that occur in major biological systems, leading to the production of free radicals that damage proteins and DNA^8^. As already mentioned, heavy metals can accumulate in different body districts, including bone tissue. Indeed, there are several studies reporting a correlation between Pb and Cd blood levels and Bone Mineral Density (BMD), with consequent increased risk of fragility fractures^9^. Absorbed Pb circulates in the plasma and then is exchanged mainly between different tissues ^10^. Bone is capable of storing Pb, which can be subsequently mobilized and can generate neurotoxic and teratogenic effects, respectively^11^. Environmental exposure to Cd can also affect bone homeostasis, leading to osteomalacia, osteoporosis and Itai-Itai diseases^12^. Therefore, it has been shown that Pb and Cd can exert direct effects on bone cells by affecting the processes of bone tissue apposition and resorption. To date, Al-Ghafari and colleagues report how treatment of osteoblast cultures with Pb and Cd reduces cell viability, impairing bioenergetic processes resulting in reduced levels of ATP produced^13^. The hypothesis of a potential involvement of heavy metals in bone tissue remodelling is also supported by the reports of Scimeca and colleagues, who observed accumulation of Pb, Cd, and Chromium (Cr) in femoral head biopsies of osteoporotic patients. The presence of these metals appeared to correlate with Sclerostin levels detected in the same tissues, suggesting the presence of a molecular link between heavy metal bioaccumulation and dysregulation of bone metabolism in osteoporosis^14^. Therefore, the aim of the present study was to analyze plasma levels of major heavy metals in osteoporotic patients (OPs) and healthy subjects (CTRs). In addition, circulating levels of heavy metals were correlated with the expression levels of genes encoding for three enzymes involved in the body's detoxification processes, including NAD(P)H quinone oxidoreductase (NQO1), Metallothionein 1E (MT1E) and Catalase (CAT). Data obtained highlight the pivotal role played by metals in osteoporosis, in association with the altered expression pattern of enzymes mainly involved in the defence against oxidative stress, which is essential for maintaining the homeostasis of the musculoskeletal system.

## Materials and methods

### Subjects

The study was approved by the Ethical Board of the Azienda Ospedaliera Universitaria “Policlinico Tor Vergata” (approval reference number #17/21). Subjects included in the study were divided into two groups: 31 OPs undergoing surgery for low-energy fragility fracture and 32 CTRs undergoing surgery for high-energy fracture. Subjects with malignancies, endocrine disorders affecting bone and mineral metabolism, autoimmune diseases and bone disorders other than primary osteoporosis, as well as those on long-term therapy with drugs that interfere with bone metabolism, sex hormone replacement therapy and antifracture and/or osteoanabolic therapies, alcohol abuse and HBV, HCV or HIV infections were excluded from the study. A questionnaire was administered to all subjects recruited in the study to collect information of their habit of smoking. All participants signed informed consent before surgery, and all experimental procedures were performed according to the Code of Ethics of World Medicine (Declaration of Helsinki).

### Clinical and biochemical parameters

Dual-energy x-ray absorptiometry (DXA) was performed with a Lunar DXA apparatus (GE Healthcare, Madison, WI, USA). Scans of the lumbar spine (L1–L4) and femur (neck and total) were performed, and BMD measurements (g/cm^2^) were recorded along with *t-scores*, with a coefficient of variation of 0.7%, according to the manufacturer's recommendations. Finally, Calcium (Ca), PTH and 25-(OH)-VitD levels were measured in fasting venous blood samples^15^. Detailed clinical characteristics of the study subjects are summarized in Table 1.

**Table 1:** Demographic, clinical and biochemical parameters of OPs and CTRs.​

Characteristics	OPs (n = 31)	CTRs (n = 32)	*p* value
Age (years)	61.70 ± 11.70	55.00 ± 16.00	*(*p* < 0.05)
BMI (g/cm^2^)	24.70 ± 4.30	27.60 ± 4.80	*(*p* < 0.05)
*t-score* L1–L4	− 1.06 ± 1.60	0.61 ± 1.19	***(*p* < 0.001)
*t-score* total femur	− 1.27 ± 0.78	0.81 ± 1.07	****(*p* < 0.0001)
*t-score* femoral neck	− 2.14 ± 0.33	0.14 ± 0.98	****(*p* < 0.0001)
BMD L1–L4	1.05 ± 0.19	1.25 ± 0.15	****(*p* < 0.0001)
BMD total femur	0.85 ± 0.10	1.12 ± 0.14	****(*p*< 0.0001)
BMD femoral neck	0.78 ± 0.08	1.06 ± 0.14	****(*p* < 0.0001)
Ca (mg/dL)	8.15 ± 0.60	8.51 ± 0.50	NS (*p*= 0.8398)
PTH (pg/mL)	83.41 ± 34.10	74.63 ± 28.63	NS (*p* = 0.3339)
25-(OH)-Vit D (ng/mL)	19.94 ± 12.68	18.89 ± 7.04	NS (*p* = 0.7718)
Smoke (%)	38	44	NS (*p* = 0.8134)

*BMI* body mass index, *BMD* bone mineral density, *PTH* parathyroid hormone; 25-(OH)-Vit D, 25-hydroxyvitamin D. *(*p* < 0.05); ***(*p* < 0.001); ****(*p* < 0.0001); NS: not significant.

### Specimen collection

Peripheral blood (4 ml) was collected in EDTA tubes the day after surgery. Blood was placed in a falcon containing Ficoll-Paque™ PLUS and centrifuged at 400×*g* for 20 min at 4 °C in a brake rotor. Subsequently, the layer appearing at the interface between plasma and sediment after centrifugation was carefully removed and inserted into a new tube. Finally, two washes were performed with 1X Phosphate-Buffered Saline (PBS) by centrifugation at 200×*g* for 10 min at 4 °C. Peripheral Blood Mononuclear Cells (PBMCs) pellet was resuspended adding 1 ml of TRIzol reagent (Thermo Fisher Scientific, Waltham, Massachusetts, USA) and immediately frozen at 80 °C until further processing.

### Analytical measurements of metals

The iCAP-RQ Inductively Coupled Plasma Mass Spectrometry (ICP-MS, Thermo Fisher, Bremen, Germany), operating with the collision Q-Cell (with He as collision gas) and the Kinetic Energy Discrimination technology (KED) to remove interferences, was used to quantified metals in 63 plasma samples. The operating parameters was reported in Table S1. The following metals were determined by ICP-MS: ^27^Al, ^52^Cr, ^55^Mn, ^59^Co, ^60^Ni, ^63^Cu, ^66^Zn, ^75^As, ^77^Se, ^95^Mo, ^111^Cd, ^202^Hg, ^208^Pb. The daily ICP-MS performances optimization was carried out using iCAP Q/RQ tune solution and iCAP Q/Qnova calibration Solution (Thermo Fisher). Multipoint calibration curve in the concentration range of 0.1–100 µg/L (R^2^ > 0.990) were constructed using stock standard solutions (CPAChem, Bogomilovo, Bulgaria). After thawing at room temperature, 0.5 mL of plasma samples was diluted tenfold with ultrapure deionized water (Millipore Simplicity, Sysmatec, Eyholz, Swiss) with 1% HNO_3_ (Nitric Acid 67–69%, Optima, Thermo Fisher) solution. Reagent blanks were prepared similarly to the samples, and every tenth sample included in the analytical batch. The internal standardization by ^73^Ge (SPEX CertiPrep, Thermo Fisher), ^89^Y and ^115^In (Alfa Aesar, Thermo Fisher) at 1 µg/L was added in the analytical solutions, reagent blanks and calibration solution. The limit of detection (LoD) was calculated as the concentration of the element that produced a signal three times higher than the standard deviation of replicated measurements of a reagent blanks. To assesses precision of the method the in-house quality control (QC) sample was analyzed during the test samples sequence. For check the accuracy, the Certified Reference Materials (CRMs) BCR-639 purchased from European Commission—Joint Research Centre Institute for Reference Materials and Measurements (EC-JRC-IRMM, Geel, Belgium) were analysed as samples. The Table S2 showed performance of the ICP-MS method on plasma samples: the recovery rates were ranged between 87% for Hg to 117% for Cr, the precision was lower than 9.26% and the method accuracy, evaluated on CRMs BCR-639, was 96.5% for Al, 110% for Se and 95.6% for Zn.

### RNA extraction and qRT-PCR analysis

0.2 mL of Chloroform was added into Eppendorf containing PBMCs and 1 mL of TRIzol reagent (Thermo Fisher Scientific, Waltham, MA, USA), then centrifuged at 12,000×*g* for 15 min at 4 °C to remove debris. The supernatant was collected, placed in a new tube, mixed with isopropyl alcohol, and centrifuged at 12,000×*g* for 10 min at 4 °C. The supernatant was discarded, and the RNA pellet was washed twice with 1 ml of 75% ethanol. The pellet was air-dried for 30 min, dissolved with 30 μL of RNase-free water, and stored at − 80 °C after spectrophotometric quantification. About 500 ng of RNA was purified and subjected to reverse transcription using the cDNA with High-Capacity Reverse Transcription kit (Thermo Fisher Scientific, Waltham, MA, USA). Realtime PCR was performed on an Applied Biosystems^®^ 7500 QuantStudio 5 Real-Time PCR System (Life Technologies; Carlsbad, CA, USA). The qPCR analysis was conducted using a PowerUP SYBR green kit (Thermo Fisher Scientific, Waltham, MA, USA) and the following cycles: 95 °C for 2 min, followed by 95 °C for 15 s and 60 °C for 1 min for 40 cycles. The sequences of primers are reported in Table S3. The relative difference of *NQO1*, *CAT* and *MT1E* gene expression between Ops and CTRs was calculated using 2^-DDCT method and normalized to *GAPDH* levels as the internal control^16^.

### Statistical analyses

Data were analyzed with GraphPad Prism 7.0 (GraphPad Software, Inc., La Jolla, CA, USA). Before applied statistical procedures, the assumptions of normality were checked for each variable using the D’Agostino-Pearson test. A non-parametric Mann–Whitney U-test was used for variables showing a skewed distribution, whereas data following a normal (Gaussian) distribution were processed with an unpaired Student’s t-test. The correlation analyses between clinical/biochemical data, qRT-PCR and iCAP-RQ were evaluated using the Spearman’s rank test. Data are presented as the mean ± standard error mean (SEM). Differences were considered significant when the *p value* was < 0.05 *, *p* < 0.01 **, *p* < 0.001 ***, *p *< 0.0001 **** ^15^.

### Ethics approval

The study was approved by the Ethical Board of the Azienda Ospedaliera Universitaria “Policlinico Tor Vergata” (approval reference number #17/21). All experimental procedures were performed according to the Code of Ethics of World Medicine (Declaration of Helsinki).

### Consent to participate

Informed consent was obtained from all individual participants included in the study.

## Results

### Clinical and biochemical characteristics of the study cohort

The study was performed in 63 subjects (OPs, n = 31; CTRs, n = 32), and their clinical and biochemical characteristics are shown in Table 1. The mean age was 61.7 ± 11.7 for the OPs and 55 ± 16 for the CTRs. The average body mass index (BMI) was lower in the Ops group than in the CTRs. DXA examination assessed bone mineral density parameters (BMD and *t-score*), which differed significantly between OPs and CTRs. Specifically, OPs showed a *t-score* at lumbar vertebrae L1–L4, total femur, and femoral neck significantly lower respect to CTRs (− 1.06 ± 1.6 vs 0.61 ± 1.19, *p* < 0.001; − 1.27 ± 0.78 vs 0.81 ± 1.07, *p* < 0.0001; − 2.14 ± 0.33 vs 0.14 ± 0.98, *p* < 0.0001). BMD values at lumbar vertebrae L1–L4, total femur, and femoral neck were also significantly lower respect to CTRs (1.05 ± 0.19 vs 1.25 ± 0.15, *p* < 0.0001; 0.85 ± 0.10 vs 1.12 ± 0.14, *p* < 0.0001; 0.78 ± 0.08 vs 1.06 ± 0.14, *p* < 0.0001). Otherwise, biochemical parameters showed no significant difference between OPs and CTRs. Finally, smoking data obtained through the self-administered questionnaire to volunteers, showed no statistically significant difference between OPs and CTRs.

### Plasma concentrations of heavy metals

**Figure 1:**
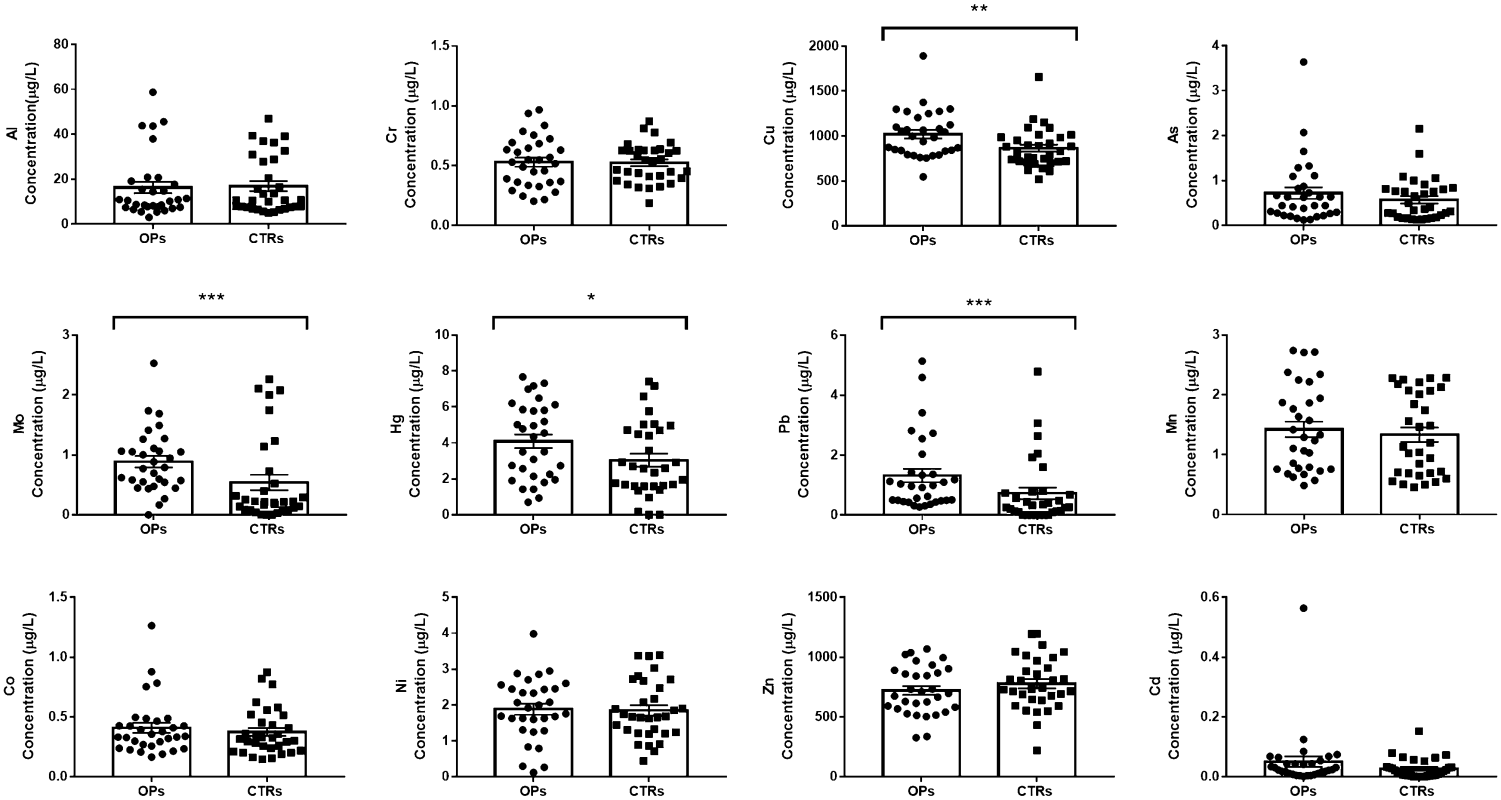
Plasma concentrations of metals (µg/L) in OPs and CTRs. Cu, Mo, Hg and Pb were found to be significantly higher in the plasma of OPs than in CTRs (*p = 0.0053, ***p = 0.0007, *p = 0.0446 and ***p = 0.0005, respectively).

In order to confirm an alteration in the levels of heavy metals related to osteoporosis, we performed circulating analysis in plasma samples of our study cohort. Plasma levels of Al, Cr, Mn, Co, Ni, Cu, Zn, As, Se, Mo, Cd, Hg, Pb were determined by ICP-MS, respectively. Our results showed that Cu, Hg, Mo and Pb plasma concentrations were significantly higher among OPs than CTRs (Fig. 1).

The average concentration of Mo and Pb in OPs was almost 2 times higher than in CTRs (0.89 ± 0.09 µg/L vs 0.54 ± 0.12 µg/L, *p* = 0.0007 and 1.32 ± 0.22 µg/L vs 0.73 ± 0.19 µg/L, *p* = 0.0005, respectively). On the other hand, the mean plasma concentration levels of Cu and Hg were about 25% and 16% higher, respectively, in the OPs than in the CTRs (1022 ± 46.85 µg/L vs 866 ± 38.99 µg/L, *p* = 0.0053 and 4.09 ± 0.38 µg/L vs 3.04 ± 0.36 µg/L, *p* = 0.0446, respectively) (Table 2).

**Table 2:** Plasma concentrations of metals (µg/L) and relative Limit of Detection (LoD) in OPs and CTRs.

Metals	Units	LoD	OPs	CTRs	*p*-value
Al	µg/L	0.170	16.31 ± 2.56	16.93 ± 2.23	NS (*p* = 0.9837)
As	µg/L	0.044	0.72 ± 0.13	0.57 ± 0.08	NS (*p* = 0.4731)
Cd	µg/L	0.001	0.05 ± 0.02	0.03 ± 0.01	NS (*p* = 0.0851)
Co	µg/L	0.001	0.41 ± 0.04	0.37 ± 0.03	NS (*p* = 0.4753)
Cr	µg/L	0.034	0.53 ± 0.04	0.52 ± 0.03	NS (*p* = 0.9115)
Cu	µg/L	0.510	1022 ± 46.85	866 ± 38.99	**(*p* = 0.0053)
Hg	µg/L	0.007	4.09 ± 0.38	3.04 ± 0.36	*(*p* = 0.0446)
Mn	µg/L	0.035	1.42 ± 0.13	1.33 ± 0.12	NS (*p* = 0.4816)
Mo	µg/L	0.042	0.89 ± 0.09	0.54 ± 0.12	***(*p* = 0.0007)
Ni	µg/L	0.016	1.88 ± 0.15	1.85 ± 0.14	NS (*p* = 0.8818)
Pb	µg/L	0.0038	1.32 ± 0.22	0.73 ± 0.19	***(*p* = 0.0005)
Zn	µg/L	0.035	720.10 ± 36.34	778.40 ± 38.98	NS (*p* = 0.2784)

*(*p* < 0.05); ** (*p* < 0.01); *** (*p* < 0.001); NS: not significant.

### Expression profile analysis of detoxifying *NQO1*, *MT1E* and *CAT* encoding genes

**Figure 2:**
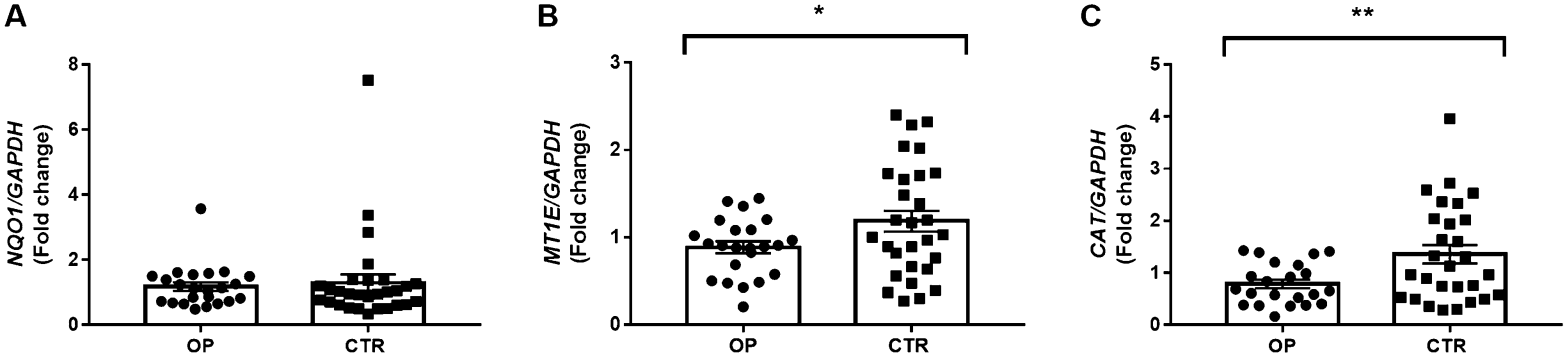
Analysis of detoxifying *NQO1*, *MT1E* and *CAT* encoding genes expression levels in PBMCs of 31 OPs and 32 CTRs. (**A**) OPs group show a slight decreased pattern in the expression level of *NQO1* compared with CTRs (NS, *p* = 0.3698). (**B**, **C**) OPs show decreased expression levels of *MT1E* and *CAT* compared with CTRs (*, *p* = 0.0349, **, *p* = 0.0059). The mRNA level of *GAPDH* was used to normalize the relative amount of detoxifying genes and the relative expression values are expressed as 2-ΔΔCT.

In order to investigate whether heavy metal exposure altered the expression levels of detoxifying genes, the mRNA expression levels of *NQO1*, *MT1E* and *CAT* in OPs group and CTRs were quantified by qRT-PCR. No significant difference was found in the *NQO1* expression levels between OPs and CTRs (1.18 ± 0.13 *vs* 1.30 ± 0.25, *p* = 0.3698). Conversely, we found a significant inhibition of *MT1E* and *CAT* mRNA expression levels in OPs compared to CTRs (0.88 ± 0.07 vs 1.18 ± 0.12, *p* = 0.0349 and 0.78 ± 0.08 vs 1.36 ± 0.18, *p* = 0.0059, respectively) (Fig. 2).

### Correlation between plasma concentration of heavy metals and PBMCs detoxifying genes expression levels

**Figure 3:**
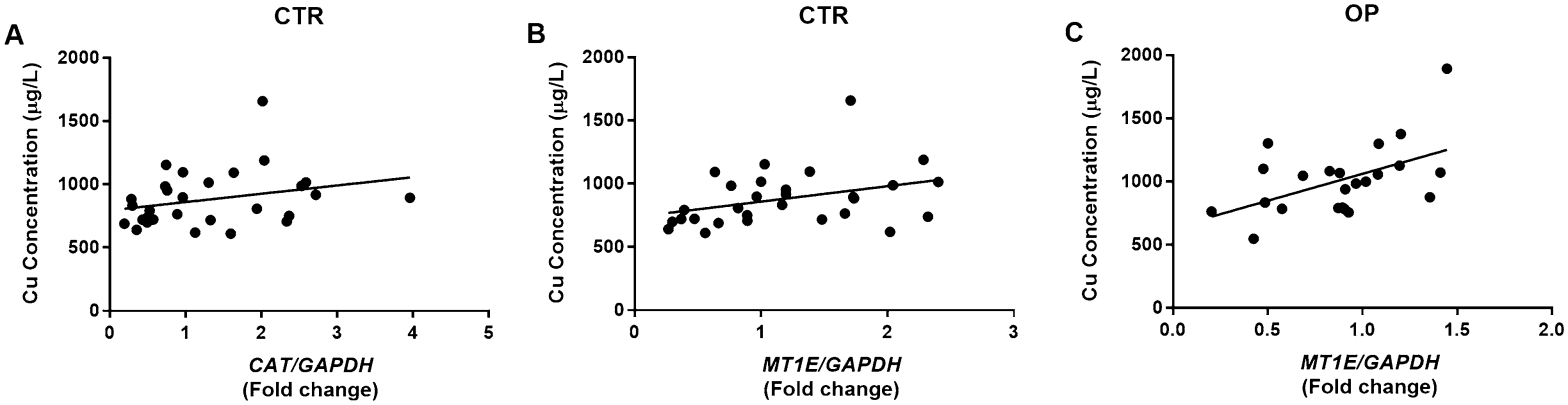
Correlation between PBMCs expression levels of *CAT *and *MT1E *genes and Cu concentration in plasma from OPs and CTRs. (**A**) *CAT *expression levels were positively associated with Cu concentration (μg/L) in CTRs. Spearman *r* = 0.3675; 95% CI = − 0.01023 to 0.6534, *p = 0.0499. (**B**, **C**) *MT1E* expression levels were positively associated with Cu concentration (μg/L) both in CTRs and OPs. CTRs showed Spearman *r* = 0.4261, 95% CI = 0.05932–0.6915, *p = 0.0212. OPs showed Spearman *r* = 0.4486, 95% CI = 0.03174–0.7325, *p = 0.0318.

To identify possible correlations between circulating levels of heavy metals and altered expression pattern of detoxifying enzymes in OPs, a correlation analysis was conducted. By applying the Spearman correlation test, a statistically significant direct correlation was identified between Cu levels and the expression levels of both *CAT* and *MTE1* in CTRs (*p* = 0.0499 and* p* = 0.0212, respectively). In addition, a statistically significant direct correlation was identified between Cu levels and *MTE1* expression in OPs (*p* = 0.0318) (Fig. 3).

## Discussion

Trace metals are ubiquitous environmental pollutants with toxic properties^17^. Human exposure to these chemicals may occur occupationally, environmentally, or through dietary intake. In the general population, the main risk factors are represented by food and water, while cigarette smoking is an additional relevant source of exposure to trace metals^18^. Contaminants in food and water enter the body through absorption by the gastrointestinal tract, as is the case of Pb, Cd, and As. Pb and As can also enter through the skin, while Cd enters the respiratory tract through inhalation^18^. The exposure of metals is capable of causing deleterious effects on all systems of the human body. Several in vivo and in vitro studies show how heavy metals can affect bone homeostasis, inhibiting skeletal growth and deposition of new bone matrix, thus leading to degenerative diseases, including osteoporosis and osteoarthritis^19^. The present work aims to identify the role of circulating metal levels in OPs, investigating their essential involvement in the pathogenesis of the disease and highlighting the need to further study their role in osteoporosis. There are growing literature data that heavy metals result in altered homeostasis of bone tissue, leading to the development of chronic degenerative diseases such as osteoporosis and osteoarthritis^9,20^. A recent study analyzed the association between the presence of toxic metals in serum and BMD in 60 male adults, showing a correlation between blood Pb and Cd levels and the risk of osteoporosis^21^. Interestingly, blood levels of both metals correlated negatively with blood levels of Ca, suggesting a potential involvement of Pb and Cd in modulating BMD and consequently representing an important risk factor for the onset of osteoporosis. Confirming the potential role played by metals in bone metabolism, Sadeghi et al*.* identified increased bioaccumulation of Zn, Cu, Pb and Cd in the plasma of 49 osteoporotic women. Although the result was not statistically significant, the differences were greater in patients with more severe disease (*t-score* < − 1.7) and the presence of the metals correlated negatively with the femur *t-score*^22^. Confirming these preliminary data, our results broaden the landscape of the role played by metals in osteoporosis by identifying higher concentrations of Cu, Mo, Hg, and Pb in the plasma of OPs compared to CTRs. It is known that excess Cu can influence generalized BMD loss, causing rickets and abnormal osteophyte formation in patients with Wilson's disease^23^. Furthermore, as reported by Qu and colleagues, elevated serum Cu levels were found to be significantly associated with increased osteoporosis fractures, especially in men, supporting our results^24^. Pb, on the other hand, represents an important environmental pollutant, and several studies have shown that acts negatively on osteoblasts and osteoclasts, delaying the healing time of fractures by inhibiting the progression of endochondral ossification^25^. In addition, it was often found that bone tissue from OPs with increased Pb presence had lower histomorphometric Bone Volume (BV/TV) values^14^. Finally, the effect exerted by bioaccumulation of Hg and Mo on bone has not yet been elucidated^23^. Although bioaccumulation of metals is now known to exert negative effects, either directly or indirectly, on bone tissue, studies investigating the potential role played by bioaccumulation of Hg on BMD are conflicting and in most do not report statistically significant correlations^26^. Wei et al*.* investigated the effect of some dietary intake metals on BMD, reporting that co-exposure to Pb, Cd, Hg, Selenium (Se), Manganese (Mn), and Zinc (Zn) correlated with decreased BMD, however, only Pb, Mn, and Se were identified as the main contributors to this association^27^. Our results also report elevated serum levels of Mo in OPs, compared to CTRs. In agreement with this finding, Lewis et al.^28^ identify an inverse association between the amount of Mo in urine and BMD of both the lumbar spine and femoral neck, suggesting that this metal may impair bone metabolism by interfering with sex steroid hormone levels. Bioaccumulation of heavy metals affects different body districts directly, by altering cell viability and function, and indirectly, such as by increasing levels of oxidative stress. Thus, the presence of high amounts of toxic metals leads to an increase in ROS, but also to the depletion of intracellular antioxidant resources and the impairment of the functionality of some detoxifying enzymes^29^. In this context, heavy metal exposure has been shown to significantly inhibit mRNA expression levels of *MT1E* and *CAT*, which represent enzymes that can defend the cell against oxidative stress^3^. Based on these data, our study aimed to investigate how levels of certain heavy metals in the circulation of OPsis related to altered expression of 3 detoxifying genes, *NQO1*, *CAT*, and *MT1E*. Our analysis shows that in OPs, the levels of metals observed in the bloodstream correlates with a deregulation of *MT1E* and *CAT* gene expression. These findings are in agreement with reports in the literature by Azizieh and colleagues, who found decreased serum levels of 5 markers of oxidative stress, including CAT, correlated with decreased BMD levels in OPs group^30^. In addition, Pb, Hg, and Cd levels have been reported to correlate with decreased *MT1* expression levels in heavy metal-exposed group, in agreement with our data^3^. Finally, further analysis identified a statistically significant direct correlation between Cu levels and *CAT* and *MTE1* expression levels in CTRs and between Cu levels and *MTE1* expression in OPs. These data show that Cu may play a predominant role in modulating the expression of both detoxifying enzymes, suggesting how this metal plays an important role in maintaining homeostasis. Overall, the results of this study broaden the landscape of knowledge on the role of heavy metals in the pathophysiology of osteoporosis. The present study presents a possible bias in the selection of individuals, as healthy subjects present a lower average age than OPs. However, this bias is not easily resolved due to the difficulty of recruiting older individuals without age-related bone diseases and other comorbidities. Further studies to be conducted on a larger cohort of OPs will be needed to validate these preliminary data. In addition, the analysis of other biological matrices, including blood and bone tissue, could better clarify the effect of metal exposure on the onset of musculoskeletal disease. Investigating the mechanisms of impact of contaminant on human health is, however, crucial, especially in association with multifactorial diseases such as osteoporosis.

## Supplementary Information

Supplementary Information.

## Data Availability

The datasets used and/or analyzed during the current study are available from the corresponding author on reasonable request.
