# Supplementary material for: Plasma heavy metal levels correlate with deregulated gene expression of detoxifying enzymes in osteoporotic patients
Source: Sci Rep. 2023 Jun 30;13:10641. doi: 10.1038/s41598-023-37410-8 (PMC10313696; doi:10.1038/s41598-023-37410-8)
Supplement: Supplementary file 1 — Supplementary Information. [file 41598_2023_37410_MOESM1_ESM.docx]

**Table S1.** Inductively Coupled Plasma Mass Spectrometry (ICP-MS) operating parameters.

| **Parameter** | **Value** |
| --- | --- |
| Spraycamber | Quartz cyclonic, cooled at 3 °C |
| Nebulizer | MicroMist borosilicate pumped at 400 µL/min |
| Injctor | 2.5 mm Quartz injector |
| Interface | Ni sample cone and insert type skimmer |
| Plasma power | 1550 W |
| Nebulizer gas | 1.1 L/min |
| Collision gas | He 4.5 mL/min |
| Kinetic Energy Discrimination (KED) | 3 V |
| Lens setting | Auto tune methods |

**Table S2**. Performance of the ICP-MS method for elements quantification in plasma

| **Metals** | **Recovery (%)** | **Precision (%)** | **Accuracy^a^**  **(%)** |
| --- | --- | --- | --- |
| Al | 101 | 7.97 | 96.5 |
| As | 111 | 2.25 | nc |
| Cd | 106 | 1.77 | nc |
| Co | 104 | 2.87 | nc |
| Cr | 117 | 4.61 | nc |
| Cu | 116 | 2.32 | nc |
| Hg | 87 | 3.43 | nc |
| Mn | 112 | 7.23 | nc |
| Mo | 107 | 9.26 | nc |
| Ni | 103 | 2.42 | nc |
| Pb | 104 | 5.67 | nc |
| Se | 113 | 2.73 | 110 |
| Zn | 113 | 1.77 | 95.6 |

^a^Calculated on the CRMs BCR-639, Human Serum

nc, not certified

**Table S3**. Primers sequences used for qRT-PCR analysis.

| ***Gene*** |  | **Sequence (5'-3')** | **Product Length (bp)** |
| --- | --- | --- | --- |
| *NQO1*  NM_000903.3 | Forward | GGTTTGAGCGAGTGTTCATAGG | 129 |
|  | Reverse | GCAGAGAGTACATGGAGCCAC |  |
| *MT1E*  NM_001363555.2 | Forward | CTCGAAATGGACCCCAACTG |  |
|  | Reverse | CAGCCCTGGGCACACTTG | 149 |
| *CAT*  NM_001752.4 | Forward | GATAGCCTTCGACCCAAGCA | 100 |
|  | Reverse | ATGGCGGTGAGTGTCAGGAT |  |
| *GAPDH*  NM_002046.7 | Forward | GATCATCAGCAATGCCTCCTG | 126 |
|  | Reverse | GTCTTCTGGGTGGCAGTGAT |  |
